# Pancreaticoduodenectomy with Anatomical Vascular Variant in Groove Pancreatitis—A Case Report

**DOI:** 10.3390/medicina60040626

**Published:** 2024-04-12

**Authors:** Gelu M. Breaza, Radu G. Dan, Florin E. Hut, Flavia Baderca, Octavian M. Cretu, Laurentiu V. Sima

**Affiliations:** 1 Department of Surgery I, “Victor Babes” University of Medicine and Pharmacy, 300041 Timisoara, Romania; gelubreaza@gmail.com (G.M.B.); florin_hut@yahoo.com (F.E.H.); tavi.cretu@yahoo.com (O.M.C.); lica_sima@yahoo.com (L.V.S.); 2 Department of Microscopic Morphology, “Victor Babes” University of Medicine and Pharmacy, 300041 Timisoara, Romania; baderca.flavia@umft.ro

**Keywords:** smoking, severe fibrosis, arterial abnormality, groove pancreatitits, pancreatoduodenectomy

## Abstract

Groove pancreatitis represents a chronic focal form of pancreatitis affecting the zone between the pancreatic head and the duodenal “C” loop, known as the groove area. This is a rare condition that affects the pancreatic periampullary part, including the duodenum and the common bile duct, which is usually associated with long-term alcohol and tobacco misuse, and is more frequent in men than in women. The most common clinical symptoms of groove pancreatitis include weight loss, acute abdominal pain, nausea, and jaundice. This report is about a 66-year-old woman with a history of heavy smoking, presenting with weight loss, nausea, and upper abdominal pain. Contrast-enhanced computed tomography revealed the existence of chronic pancreatitis as well as the dilatation of the main pancreatic duct, a cyst of the pancreatic head, and enlargement of the biliary tract. Conservatory treatment was initiated but with no improvement of symptoms. Since endoscopic retrograde cholangiopancreatography was not possible due to the local changes, we decided to perform pancreatoduodenectomy, as surgery appears to be the single effective treatment.

## 1. Introduction

Groove pancreatitis is defined as chronic segmental pancreatitis involving the duodenum and the pancreas [1]. It is most frequently associated with alcohol and tobacco misuse and is significantly more prevalent in men than in women. This is an unusual form of chronic segmental pancreatitis that affects the anatomical space between the head of the pancreas, the duodenum, and the common bile duct [2]. This rare but distinct entity is often clinically confused with ampular neoplasia, tumors of the duodenum, cystic tumors of the pancreatic head, and acute relapsing pancreatitis. The pathogenesis of groove pancreatitis is thought to be an anatomical or functional obstruction of the papilla [3]. The viscosity of the pancreatic juice increases due to heavy alcohol consumption and smoking. In the groove area, severe fibrosis and scarring occur. Characteristic pathological findings include cystic lesions in the duodenal wall, Brunner gland hyperplasia, dilatation of Santorini’s duct, and protein plaques in the pancreatic duct [4]. The efficacy of conservative treatment using endoscopic stenting is controversial. Most authors agree that pancreatoduodenectomy is the most effective treatment method for symptomatic groove pancreatitis [4,5].

## 2. Detailed Case Description

The patient is a 66-year-old woman, with a medical history of cardiovascular disease (high blood pressure, coronary artery disease, previous myocardial infarction, mitral, and tricuspid valve regurgitation) and combined hyperlipidemia. She presented to the emergency department with acute pain in the right hypochondriac and in the epigastric region that radiates in the back. The patient had a body mass index of 25 kg/m^2^ and was a heavy smoker (more than 20 cigarettes per day).

**Figure 1:**
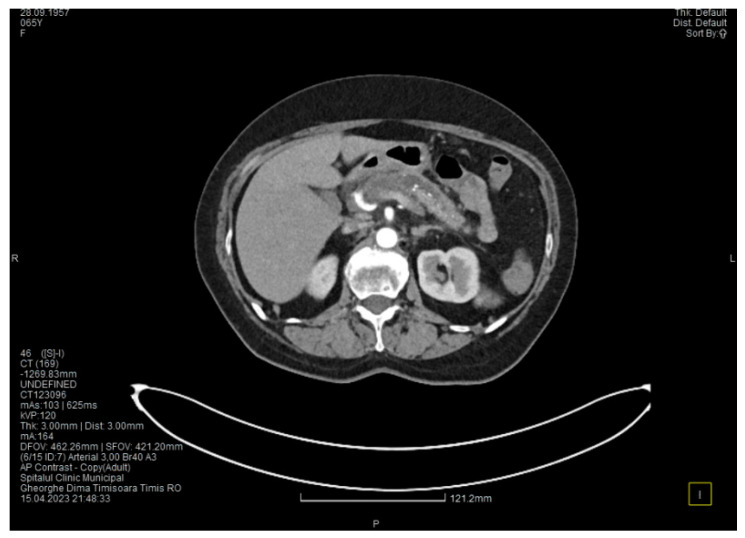
The initial CT scan shows a dilated Wirsung duct and calcifications in the pancreatic tissue.

**Figure 2:**
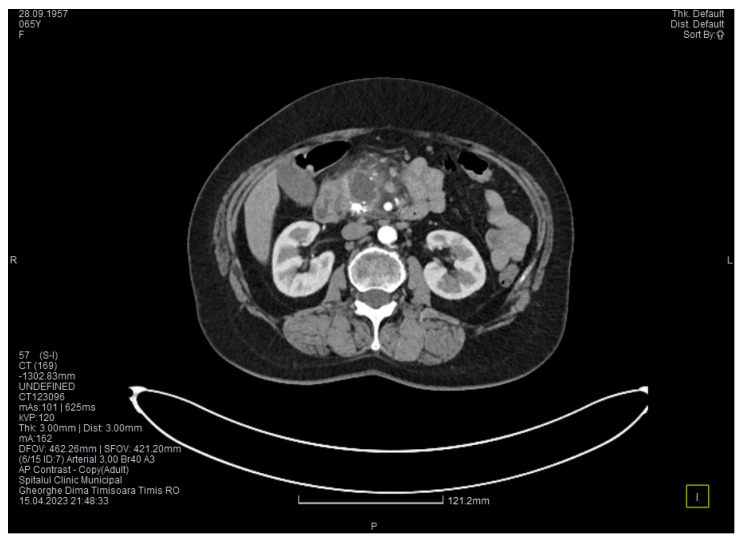
Initial CT scan shows a cyst in the pancreatic head.

Laboratory tests showed a serum lipase level more than 10 times above the upper normal range. The contrast-enhanced computed tomography (CT) showed multiple calcifications in the pancreatic tissue, dilatation of the main pancreatic duct measuring approximately 12.5 mm (Figure 1), a non-iodophilic intraparenchymal cyst in the head of the pancreas measuring 23/25 mm, which was associated with acute inflammation (Figure 2), and densification of the peripancreatic fat, which was more intense at the head of the pancreas. In addition, enlarged pericephalic and periaortocaval lymph nodes were observed. There were no changes in the liver or the gallbladder, only a mild ectasia of the intrahepatic bile ducts, and no dilatation of the common bile duct. Other changes included multiple atheromas of the abdominal aorta. The symptoms, the lab results, and the CT findings led to the diagnosis of acute pancreatitis.

We initiated treatment with antispasmodic and antisecretory drugs, as well as fluid replacement, followed by an improvement of the symptoms and lab results. The patient was discharged after 5 days.

Approximately one month later, the patient presented again to the emergency department with complaints of epigastric pain, fatigue, and malaise. The lab results were unremarkable, except for a slight increase in serum lipase (about 1.5 times higher than the upper normal range).

**Figure 3:**
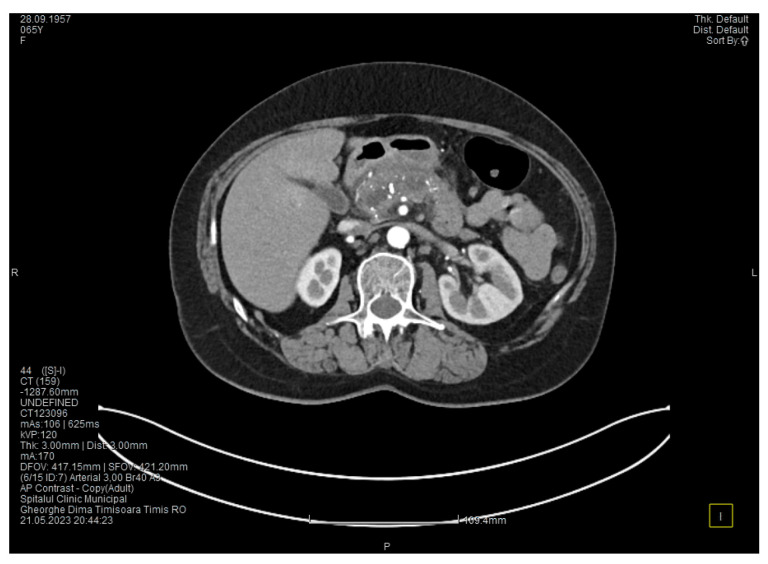
The second CT evaluation shows a dilated Wirsung duct and calcifications in the pancreatic tissue.

**Figure 4:**
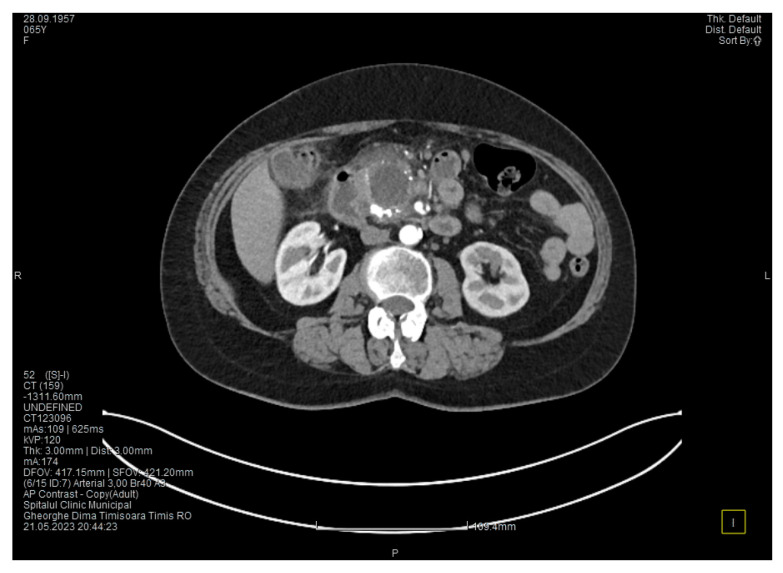
The second CT scan shows a cyst in the pancreatic head and calcifications in the pancreatic head and groove area.

The contrast-enhanced computed tomography showed changes suggestive of chronic pancreatitis, with multiple very small calcifications of the pancreatic tissue, dilatation of the Wirsung duct in the corporeo-caudal area measuring approximately 15 mm (Figure 3), and a narrowing of the Wirsung tract in the cephalic area where calcareous conglomerates were present. In the anterior cephalo-uncinate area, an oval cyst measuring 31/28 mm was detected (larger than it was one month ago) (Figure 4). In the peri-cephalo-uncinate area, we observed densifications of the adjacent fat extending towards the gastric antrum, the root of the mesentery, and the hepatic flexure of the colon. There was a mild inflammatory enlargement of the peripancreatic lymph nodes and the ones situated in the hepatic hilum. We also noticed a slight dilatation of the intrahepatic bile ducts and the common hepatic duct, and diffuse atheromatosis of the aorta, the iliac, and the common hepatic arteries. We performed a gastroscopy, which revealed a normal esophagus, stomach, and duodenal bulb; however, it was impossible to advance the endoscope towards the D2 part of the duodenum due to the significant edema of the duodenal mucosa and the partial stenosis of the duodenal lumen.

We initiated treatment with analgesic and antisecretory drugs, along with an adequate diet, resulting in the improvement of the symptoms and the discharge of the patient.

One month later, the patient returned to the emergency department complaining of progressive abdominal pain radiating to the lumbar area, asthenia, and fatigue. The laboratory tests confirmed a new episode of acute pancreatitis (increased serum amylase and lipase—about 7 times higher than the upper normal range), with an elevated C reactive protein, increased alkaline phosphatase, and hipopotassemia.

The contrast-enhanced computed tomography showed no major changes compared to the one performed one month ago, except for a more intense peripancreatic inflammation (mainly in the cephalic area).

We initiated treatment with analgesic, antispasmodic, and antisecretory drugs and performed hydroelectrolytic rebalancing, with remission of the symptoms and discharge.

Four months after this episode, the patient presented again in the emergency department complaining of pain in the upper abdomen and postprandial vomiting, symptoms that started approximately 4 weeks ago. During the clinical examination, the abdomen was tender on palpation in the epigastric and the right hypochondriac area, with no signs of peritoneal irritation.

The lab results showed a slight increase in serum amylase and lipase levels (about 2 times higher than the upper normal range) and a mild elevation of transaminases and blood glucose.

**Figure 5:**
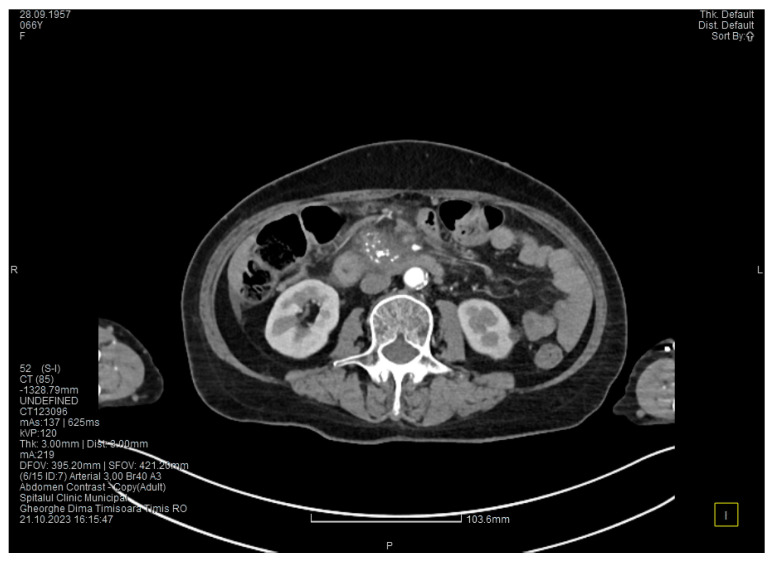
CT scan at the fourth episode of acute pancreatitis shows calcifications in the pancreatic head, thickening of the duodenal wall, and partial occlusion of the duodenum.

**Figure 6:**
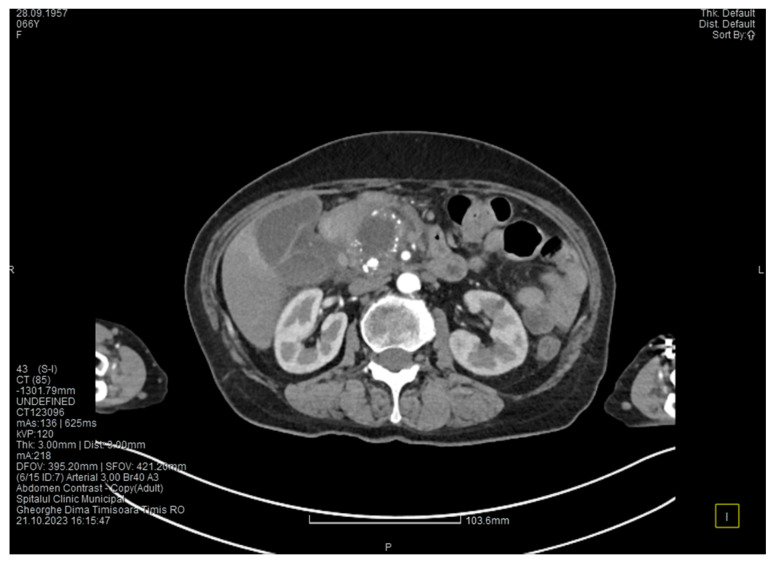
CT scan at the fourth episode of acute pancreatitis shows a slightly increased cyst and calcifications in the pancreatic head.

Contrast-enhanced computed tomography highlighted signs of chronic pancreatitis with multiple calcifications throughout the pancreatic parenchyma, calcareous conglomerates in the cephalic area (Figure 5), dilatation of the Wirsung duct measuring approximately 15 mm, a cephalo-uncinate pseudocyst measuring 30/31/32 mm (Figure 6), and densification of the pericephalic pancreatic fat; all the changes were more pronounced compared to the previous examinations. Other findings included acute cholecystitis with thickening of the gallbladder wall and iodophilia of the mucosa, small dilatations of the intrahepatic bile ducts, fluid accumulation in the hepatic hilum and in the periduodenal area, inflammatory wall thickening in the gastric antro-pyloric region and in the duodenum (I, II), and inflammatory lymph nodes in the peripancreatic area and in the hepatic hilum measuring up to 11 mm.

The CT scan showed that the pancreatic changes evolved, with an increase in the cephalic cyst and in the diameter of the pancreatic and biliary ducts. These findings suggested the diagnosis of groove pancreatitis since the changes in the pancreas were localized in the head and are extended towards the duodenum; however, this disease is quite rare in women [5]. Since most authors consider that the endoscopic retrograde cholangiopancreatography is challenging and potentially risky, and because in our case the endoscopy was technically difficult due to the local changes, we decided to perform cephalic duodenopancreatectomy, a surgical procedure recommended by most authors.

Another interesting finding before the surgery was a slight increase in CA 19-9 (1.5 times higher than the upper normal range).

The surgical procedure was represented by cephalic pancreaticoduodenectomy with continuous loop reconstruction, which involves end-to-end pancreatico-jejunal anastomosis through intussusception, end-to-side choledocho-jejunoanastomosis, prosthetic with a Kehr tube and Rachel-Polya-type gastrojejunoanastomosis with Braun anastomosis at the base of the loop.

During the surgery, we noted that the duodenum and the antro-pyloric region were pushed forward, and we found wall thickening, inflammation, and partial stenosis of the duodenal lumen. This was caused by a cephalo-pancreatic cyst with a diameter of approximately 4 cm. Subsequently, the duodenal dissection was very difficult. We also found inflammation in the hepatic hilum, causing fibrosis, and multiple local adenopathies with a diameter of up to 1 cm. The cystic duct appeared long and dilated, with a parallel path to the main bile duct, and it merged with the main bile duct in the retroduodenal area. The main bile duct presented a normal diameter (7 mm). An interesting finding was that the common hepatic artery did not originate as usual from the celiac trunk but from the superior mesenteric artery and it was partially located in the retropancreatic area. The gallbladder showed important distension, a thin wall, and no gallstones. The pancreas presented with fibrosis and multiple calcifications disseminated throughout the entire parenchyma, mainly in the groove area. The superior mesenteric vein was pulled far to the left by the inflammation, which also affected the transverse mesocolon. When sectioning the pancreatic parenchyma at the isthm, a marked dilatation of the Wirsung duct (1.5 cm) was found.

The harvested specimen was fixed in 10% (*v*/*w*) neutral buffered formalin and sent to the Department of Pathology for a microscopic examination. 

The gross examination revealed a cystic lesion measuring 1.1 cm, with smooth walls on the upper edge of the resection specimen. The duodenal mucosa had an elastic polypoid proliferation of 0.4 cm. At a distance of 1.2 cm from it, a firm nodular area of 0.6 cm was identified. At the level of the pancreatic head, numerous cystic areas were present, some with calcified content and others with brown, afractuous walls. The largest cyst had a diameter of 3 cm, along with extensive areas of fibrosis. Four lymph nodes between 0.5 and 1.1 cm were identified in the peripancreatic tissue.

Four-micrometer-thick serial sections were performed for the diagnosis from paraffin blocks, using morphological Hematoxylin–Eosin staining.

**Figure 7:**
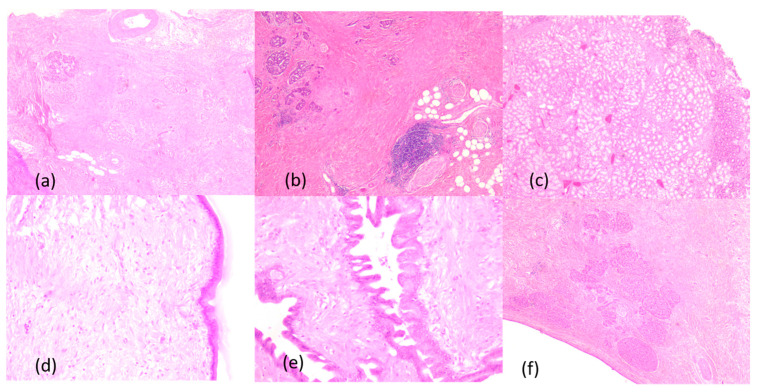
Microscopic aspects, Hematoxylin–Eosin staining: (**a**) atrophic pancreatic parenchyma with extensive areas of fibrosis, ob.5x; (**b**) fibrosis and moderate chronic inflammatory infiltrate, ob.5x; (**c**) duodenal wall with Brunner gland hyperplasia, ob.5x; (**d**) cystic lesion with low-grade pancreatic intraepithelial neoplasia (PanIn 1), ob.5x; (**e**) cystic lesion with high grade pancreatic intraepithelial neoplasia (PanIn 2); ob.20x; (**f**) pancreatic parenchyma with fibrosis, atrophic acini, cyst lesion and Langerhans islet hyperplasia, ob.5x.

The anatomopathologic examination of the resected piece revealed advanced chronic pancreatitis, with extensive areas of peri- and intralobular fibrosis replacing the pancreatic parenchyma, atrophic acini, Langerhans islet hyperplasia, and numerous dystrophic calcifications in the interstitium. The duodenal wall presented Brunner gland hyperplasia and moderate myofibroblastic proliferation. Also, there were ductal changes with distortion, ectasia, and foci of low- and high-grade pancreatic intraepithelial neoplasia (PanIn 1 and PanIn 2), characterized by columnar mucin-producing cells, nuclear enlargement, nuclear crowding, pseudostratification, nuclear hyperchromatism and a few typical mitoses. The cystic lesions also included a cyst with mucinous neoplasia composed of cells containing intracytoplasmic mucin and hypercellular ovarian-type stroma. The anatomopathological examination of the lymph nodes showed only chronic reactive lymphadenitis. The main histopathological aspects are presented in Figure 7.

After surgery, the outcome of the patient was favorable, with no complications, allowing a discharge nine days after surgery.

Groove pancreatitis is quite a rare disorder, but the case we presented shows some individualizing particularities. The first one is that our patient with groove pancreatitis was a woman with tobacco but no alcohol misuse. Groove pancreatitis is more frequent in men, usually with a history of heavy smoking and alcohol misuse. The second particularity is the anatomical abnormality of the common hepatic artery that originated from the superior mesenteric artery and was partially situated behind the pancreas. And the third one is that the anatomopathologic examination showed signs of malignancy, which is not common in groove pancreatitis.

## 3. Discussion

Described for the first time as a separate entity by Stolte et al. in 1982 [6], groove pancreatitis remains a condition rarely described in the literature. Between 1990 and 2022, only 1404 cases were presented [5]. It mainly affects males in their fifth and sixth decades of life, with a history of alcohol misuse and heavy smoking. It is associated with anatomical changes in the duodeno-pancreatic area, such as cystic dysplasia of the heterotopic pancreas, stenosis of the Santorini duct, and Brunner gland hyperplasia [4]. Our case involved a female patient in her seventh decade of life, with a long history of heavy smoking and significant weight loss in the last 6 months. The CT scan presented cystic dysplasia in the head of the pancreas and diffuse pancreatic calcifications, localized mainly in the pancreatic head. The patient’s symptomatology was characteristic of groove pancreatitis, presenting with recurrent upper abdominal pain, nausea, and vomiting, most likely in the context of secondary duodenal stenosis.

Groove pancreatitis is classically divided into two forms: the pure form, which strictly affects the groove space, and the segmental form, in which the changes affect the groove space but extend medially and to the pancreatic head [2]. In some cases, such as in ours, the changes in chronic pancreatitis can extend to the entire pancreatic parenchyma, a situation revealed by imaging and the result of the histological examination.

Groove pancreatitis is still an underdiagnosed condition with an unknown real incidence. Recently, improvements in imaging techniques and the publication of radiological criteria have corroborated the recognition of symptoms, as evidenced by the multitude of studies dedicated to the diagnosis and treatment of this condition in recent years. The incidence of groove pancreatitis varies between 2.7% and 24.5% in pancreaticoduodenectomies performed for chronic pancreatitis [7]. In a study conducted on 600 patients resected for chronic pancreatitis, Becker et al. found various degrees of involvement of the groove area in 19.5% of the cases; the pure form was found in 2% of patients, the segmental form in 6.5%, and 11% of the patients showed lesions in the entire pancreatic parenchyma [8]. 

Conservative treatment, in most cases, is the first-line treatment and is represented by the administration of analgesics, proton pump inhibitors, somatostatin derivatives, pancreatic enzymes, an adequate diet, and proper hydration. This approach, according to the published data, may lead to a complete remission of symptoms in only one-third of the cases [2], with frequent episodes of recurrence. In our presented case, conservative treatment led to the improvement of the patient’s symptoms, but only for short periods of time, followed by frequent relapses that required several hospitalizations in the last 6 months. In addition to the impossibility of excluding a malignant degeneration, this led to the necessity for surgical treatment.

Endoscopic treatment, whenever possible, is a treatment option for patients with groove pancreatitis as an alternative to surgical treatment, given the fact that it is less invasive and has lower morbidity. The results of this approach vary from one study to another. While some studies show a success rate of approximately 50%, with relapses in more than half of the patients [2,9,10], others show complete remission in 70% of the cases [11,12]. Bender et al., in a study conducted on seven patients who underwent endoscopic retrograde cholangiopancreatography, reported extremely poor results, with none of the patients presenting a complete remission of symptoms at 6 months, and five of them requiring surgical intervention [13]. For the patient we presented, the gastroenterologist of our team evaluated the situation and decided that the ERCP procedure was not possible.

Surgery remains an important therapeutic approach in groove pancreatitis, especially in cases that do not respond to conservative treatment or in which pancreatic carcinoma cannot be excluded [12]. The procedure of choice for groove pancreatitis is pancreaticoduodenectomy, although there are studies in the literature that report alternative procedures but with contradictory results. Egorov et al., in a study conducted on 84 patients with groove pancreatitis, reported superior results of duodenal resection with pancreas preservation compared to pancreaticoduodenectomy, with a complete remission of symptoms in 93% of cases, compared to 84% in pancreaticoduodenectomy [14]. The motivation for this type of surgical treatment is that pancreaticoduodenectomy is too extensive to treat the pure form of groove pancreatitis, which is the least frequent form. Moreover, this approach cannot exclude pancreatic malignant degeneration if this suspicion exists. Duodenal or biliary bypass interventions can be useful in case of stenoses [15], but they address only the complications of pancreatitis and will have limited effects on symptom control. Pancreaticoduodenectomy, with or without pylorus preservation, represents the most commonly used technique for the treatment of groove pancreatitis, presenting a major advantage over all other procedures in which malignant degeneration cannot be excluded. The results of pancreaticoduodenectomy are obviously better than those obtained by conservative or endoscopic treatment, with a high percentage of total remission [4,5,14,16]. Although it shows the best results, pancreaticoduodenectomy is a major surgical procedure, with mortality at 90 days reaching 5% [17] and postoperative morbidity that reaches 45% [18]. Moreover, the changes produced by the pancreatic and peripancreatic inflammation, which make dissection much more difficult, can lead to an increased rate of postoperative complications. Considering the fact that a long duration of groove pancreatitis leads to severe inflammation around the pancreas, the best approach would be to perform pancreaticoduodenectomy as early as possible in cases of conservative treatment failure, thus avoiding a potential increase in the risk of complications.

The main challenge in groove pancreatitis is the differentiation from pancreatic ductal adenocarcinoma. Although this differentiation can be extremely difficult without an anatomopathologic examination, Ukegjini et al., in a review including 1404 patients, found some significant criteria that advocate groove pancreatitis. The factors suggesting groove pancreatitis are young age, male gender, a history of alcohol misuse and heavy smoking, presentation with pain without jaundice, and inflammatory and/or cystic changes in the duodenal wall [5]. Moreover, one-third of the patients with groove pancreatitis showed elevated levels of carcinoembryonic antigen and CA 19-9 [19], making precise diagnosis more difficult since there is no threshold for these lab tests that could allow the differentiation between the two conditions. The difficulty in differentiating between the two entities questions the option for conservative treatment, knowing that patients with pancreatic adenocarcinoma have a poor prognosis if surgical treatment is delayed. This issue also raised difficulties in the differential diagnosis in our case, where, according to the anatomopathological examination, the characteristics of chronic pancreatitis and neoplasia coexist.

Although there are hundreds of case reports and studies referring to groove pancreatitis (142 case reports only in the Pub Med database), the coexistence of groove pancreatitis and malignant lesions is rarely presented in the literature. According to our knowledge, this is the second case of groove pancreatitis concurrent with malignant lesions. In 2022, Lugo-Fagundo et al. presented a 46-year-old male patient with groove pancreatitis and intraductal papillary mucinous neoplasm [20]. Based on the lack of data covering this aspect, we strongly recommend reporting all these situations that could change the management of patients with groove pancreatitis. This association of lesions, which is impossible or at least very difficult to diagnose without a surgically extracted pathological sample, also questions a more conservative approach for patients with this condition. The clinical consequences of misdiagnosis of pancreatic cancer are quite profound, and due to the frequent overlap of the clinical, laboratory, and imaging findings, a healthy skepticism of the diagnosis must always be considered [21]. Endoscopic ultrasound (EUS)-guided fine needle aspiration (FNA) has proven to be an efficient method for diagnosing pancreatic cancer [22], but when negative, one must always consider the possibility of a sampling error [21].

## 4. Conclusions

Pancreaticoduodenectomy represents an important therapeutic option in patients with groove pancreatitis in cases of conservative treatment failure, especially if pancreatic cancer cannot be excluded. If recommended, surgical treatment should be performed as early as possible, in order to avoid the extension of the inflammation and the subsequent formation of fibrosis, which could complicate the dissection and increase the risk of intra- and postoperative complications. The possibility of the coexistence, as in our presented case, of groove pancreatitis with neoplasia of the pancreatic head, without previous clinical, biological, and imaging findings suggestive of malignancy, is a major determinant in the recommendation to consider pancreaticoduodenectomy as the treatment of choice in patients with groove pancreatitis. We strongly recommend reporting all cases of groove pancreatitis associated with malignancy, as this could lead to unanimously accepted changes in the treatment strategies for these patients.

## Data Availability

The original contribution presented in the study are included in the article, further inquiries can be directed to the corresponding author.

## References

[1] Hernandez-Jover D., Pernas J.C., Gonzalez-Ceballos S., Lupu I., Monill J.M., Pérez C. (2011). Pancreatoduodenal Junction: Review of Anatomy and Pathologic Conditions. J. Gastrointest. Surg..

[2] Raman S.P., Salaria S.N., Hruban R.H., Fishman E.K. (2013). Groove Pancreatitis: Spectrum of Imaging Findings and Radiology-Pathology Correlation. Am. J. Roentgenol..

[3] Addeo G., Beccani D., Cozzi D., Ferrari R., Lanzetta M.M., Paolantonio P., Pradella S., Miele V. (2019). Groove pancreatitis: A challenging imaging diagnosis. Gland. Surg..

[4] Tezuka K., Makino T., Hirai I., Kimura W. (2010). Groove pancreatitis. Dig. Surg..

[5] Ukegjini K., Steffen T., Tarantino I., Jonas J.P., Rössler F., Petrowsky H., Gubler C., Müller P.C., Oberkofler C.E. (2023). Systematic review on groove pancreatitis: Management of a rare disease. BJS Open.

[6] Stolte M., Weiss W., Volkholz H., Rösch W. (1982). A special form of segmental pancreatitis: “groove pancreatitis”. Hepatogastroenterology.

[7] Dhali A., Ray S., Ghosh R., Misra D., Dhali G.K. (2022). Outcome of Whipple’s procedure for Groove pancreatitis: A retrospective cross-sectional study. Ann. Med. Surg..

[8] Becker V., Mischke U. (1991). Groove pancreatitis. Int. J. Pancreatol..

[9] Kager L.M., Lekkerkerker S.J., Arvanitakis M., Delhaye M., Fockens P., Boermeester M.A., van Hooft J.E., Besselink M.G. (2017). Outcomes After Conservative, Endoscopic, and Surgical Treatment of Groove Pancreatitis: A Systematic Review. J. Clin. Gastroenterol..

[10] Chantarojanasiri T., Isayama H., Nakai Y., Matsubara S., Yamamoto N., Takahara N., Mizuno S., Hamada T., Kogure H., Koike K. (2018). Groove Pancreatitis: Endoscopic Treatment via the Minor Papilla and Duct of Santorini Morphology. Gut Liver.

[11] Arvanitakis M., Rigaux J., Toussaint E., Eisendrath P., Bali M.A., Matos C., Demetter P., Loi P., Closset J., Deviere J. (2014). Endotherapy for paraduodenal pancreatitis: A large retrospective case series. Endoscopy.

[12] Ray S., Ghatak S., Misra D., Dasgupta J., Biswas J., Khamrui S., Bandyopadhyay D., Ghosh R. (2017). Groove Pancreatitis: Report of Three Cases with Brief Review of Literature. Indian J. Surg..

[13] Bender M.T., Sauer B., Shami V.M. (2018). Groove Pancreatitis: Management and Outcome of Patients at a Tertiary Care Center. Am. J. Gastroenterol..

[14] Egorov V., Petrov R., Schegolev A., Dubova E., Vankovich A., Kondratyev E., Dobriakov A., Kalinin D., Schvetz N., Poputchikova E. (2021). Pancreas-preserving duodenal resections vs pancreatoduodenectomy for groove pancreatitis. Should we revisit treatment algorithm for groove pancreatitis?. World J. Gastrointest. Surg..

[15] Ioannidis A., Menni A., Tzikos G., Ioannidou E., Makri G., Vouchara A., Goulas P., Karlafti E., Psoma E., Mavropoulou X. (2023). Surgical Management of Groove Pancreatitis: A Case Report. J. Pers. Med..

[16] Casetti L., Bassi C., Salvia R., Butturini G., Graziani R., Falconi M., Frulloni L., Crippa S., Zamboni G., Pederzoli P. (2009). “Paraduodenal” Pancreatitis: Results of Surgery on 58 Consecutives Patients from a Single Institution. World J. Surg..

[17] Narayanan S., Martin A.N., Turrentine F.E., Bauer T.W., Adams R.B., Zaydfudim V.M. (2018). Mortality after pancreaticoduodenectomy: Assessing early and late causes of patient death. J. Surg. Res..

[18] Giuliano K., Ejaz A., He J. (2017). Technical aspects of pancreaticoduodenectomy and their outcomes. Chin. Clin. Oncol..

[19] Tarvainen T., Nykänen T., Parviainen H., Kuronen J., Kylänpää L., Sirén J., Kokkola A., Sallinen V. (2020). Diagnosis, natural course and treatment outcomes of groove pancreatitis. HPB.

[20] Lugo-Fagundo E., Weisberg E.M., Fishman E.K. (2022). Pancreatic cancer in patient with groove pancreatitis: Potential pitfalls in diagnosis. Radiol. Case Rep..

[21] Patel B.N., Jeffrey R.B., Olcott E.W., Zaheer A. (2020). Groove pancreatitis: A clinical and imaging overview. Abdom. Radiol..

[22] Bergeron J.P., Perry K.D., Houser P.M., Yang J. (2015). Endoscopic ultrasound-guided pancreatic fine-needle aspiration: Potential pitfalls in one institution’s experience of 1212 procedures. Cancer Cytopathol..

